# Emerging Biochemical Conversion for Plastic Waste Management: A Review

**DOI:** 10.3390/molecules30061255

**Published:** 2025-03-11

**Authors:** Zhongchuang Liu, Siu Hua Chang, Gilles Mailhot

**Affiliations:** 1 Department of Environmental Engineering Technology, College of Power Engineering, Chongqing Electric Power College, No. 9, Electric Power Fourth Village, Jiulongpo District, Chongqing 400053, China; 2 Waste Management and Resource Recovery (WeResCue) Group, Chemical Engineering Studies, College of Engineering, Universiti Teknologi MARA, Cawangan Pulau Pinang, Permatang Pauh 13500, Penang, Malaysia; shchang@uitm.edu.my; 3 Institut de Chimie de Clermont-Ferrand, Université Clermont Auvergne—Centre National de la Recherche Scientifique (CNRS), F-63000 Clermont-Ferrand, France

**Keywords:** plastic waste, conversion, catalyst, mechanism, circular economy

## Abstract

In recent years, vast amounts of plastic waste have been released into the environment worldwide, posing a severe threat to human health and ecosystems. Despite the partial success of traditional plastic waste management technologies, their limitations underscore the need for innovative approaches. This review provides a comprehensive overview of recent advancements in chemical and biological technologies for converting and utilizing plastic waste. Key topics include the technical parameters, characteristics, processes, and reaction mechanisms underlying these emerging technologies. Additionally, the review highlights the importance of conducting economic analyses and life cycle assessments of these emerging technologies, offering valuable insights and establishing a robust foundation for future research. By leveraging the literature from the last five years, this review explores innovative chemical approaches, such as hydrolysis, hydrogenolysis, alcoholysis, ammonolysis, pyrolysis, and photolysis, which break down high-molecular-weight macromolecules into oligomers or small molecules by cracking or depolymerizing specific chemical groups within plastic molecules. It also examines innovative biological methods, including microbial enzymatic degradation, which employs microorganisms or enzymes to convert high-molecular-weight macromolecules into oligomers or small molecules through degradation and assimilation mechanisms. The review concludes by discussing future research directions focused on addressing the technological, economic, and scalability challenges of emerging plastic waste management technologies, with a strong commitment to promoting sustainable solutions and achieving lasting environmental impact.

## 1. Introduction

Plastic products have permeated nearly every aspect of human life due to their numerous advantages, such as corrosion resistance, lightweight properties, antibacterial features, ease of processing, and low cost. Plastics are generally categorized into two main types based on their physical and chemical properties: thermosetting plastics and thermoplastics. Thermosetting plastics include phenolic resin (PF), urea–formaldehyde resin (UF), melamine resin (MF), unsaturated polyester resin (UPR), epoxy resin (EP), silicone resin (SI), and polyurethane (PU). These plastics undergo chemical reactions and harden after being heated, pressurized, or combined with hardeners for some time. Conversely, thermoplastics, which are plastics that retain plasticity at certain temperatures, include polyethylene (PE), polypropylene (PP), polyvinyl chloride (PVC), polystyrene (PS), polyoxymethylene (POM), polycarbonate (PC), polyamide (PA), acrylic resin (PSA), polyethylene terephthalate (PET), polysulfone (PSF), acrylonitrile butadiene styrene copolymer (ABS), and polyphenylene ether (PPE). From 1950 to 2020, global annual plastic production skyrocketed, rising from 1.7 × 10^6^ t to an astonishing 3.7 × 10^8^ t [1]. This rapid growth was notably amplified in 2020, fueled by the sharp increase in the production and widespread use of medical protective equipment rich in plastics in response to the COVID-19 pandemic [2]. According to statistics, about half of the world’s plastic products become waste and enter the environment annually [3].

Plastic waste is notoriously resistant to degradation and can remain in the natural environment for extended periods. Untreated plastic waste can adversely impact water, air, and soil ecosystems. Over time, physical, chemical, or microbial interactions can degrade plastic waste into smaller fragments, including microplastics (<5 mm) and nanoplastics (<1 μm), which pose biological toxicity risks to organisms [4,5,6]. In addition, microplastic and nanoplastic particles have a large specific surface area and strong adsorption affinity, enabling them to act as carriers for heavy metals and persistent organic pollutants [7,8,9,10,11]. These characteristics make them particularly harmful to humans and ecosystems [7,8,10,11]. Therefore, the efficient conversion and reuse of plastic waste have become an urgent global priority.

Landfill and incineration are the traditional technologies for treating plastic waste [12,13]. To a certain extent, traditional technologies can solve the problem of plastic pollution. However, landfills and incineration also have many drawbacks in terms of treating plastic waste. For example, landfill methods require extensive land resources and prolonged degradation [14]. The incineration method, on the other hand, emits smoke containing toxic and harmful substances such as heavy metals, organic compounds, acidic gases, and particulate matter [14]. Additionally, the slag and fly ash generated by incineration contain heavy metals, and fly ash also contains dioxins and a large number of salts, which are classified as hazardous waste [14]. Moreover, these conventional methods fail to recover valuable resources effectively from plastic waste, emphasizing the need for innovative alternatives. Emerging technologies for plastic waste treatment are thus essential to overcome the limitations of traditional approaches.

This review aims to provide a comprehensive overview of the technical parameters, characteristics, processes, and reaction mechanisms of different emerging technologies, focusing on chemical and biological methods for converting and utilizing plastic waste. It also incorporates an economic analysis and life cycle assessment, offering valuable insights to guide future research and development. Based on recent literature, particularly from the last five yearsl across various databases, this review provides a comprehensive summary of the latest advancements in plastic waste management. It introduces a broader range of technologies than comparable reviews and uniquely integrates economic and life cycle assessments, highlighting sustainability and long-term impact.

## 2. Emerging Technologies

The technologies for converting or recycling plastic waste typically include physical, chemical, and biological methods. However, in recent years, research has increasingly focused on chemical and biological technologies, as these methods demonstrate superior efficiency and sustainability in tackling the challenges of plastic waste management [14,15,16].

### 2.1. Chemical Methods

The principle of chemical methods is to break down high-molecular-weight macromolecules in plastics into smaller oligomers or monomers through cracking or depolymerizing chemical groups within the plastic polymers. Not only does this process reduce plastic waste, but it also generates valuable chemicals that can be repurposed for various applications. Typically, the reactions of this process occur under specific conditions, such as the presence of additional reactants or catalysts, controlled temperature, reaction medium, or illumination.

Recent advancements in chemical recycling technologies of plastic waste have incorporated various devices, including tank reactors, electrolyzers, autoclaves, reaction vessels, fluidized bed reactors, furnaces, fixed bed reactors, tube reactors, pyrolyzers, microwave ovens, fluidized bed gasifiers, and tar-cracking reactors, to optimize the conversion process (Table 1). Most researchers have used thermal chemical methods, such as pyrolysis, gasification, and hydrothermal gasification, for treating plastic waste. As a result, the conversion process typically occurs at elevated temperatures ranging from 200 to 850 °C (Table 1). Pyrolysis is usually conducted in an inert gas environment (e.g., N_2_, Ar), whereas gasification can occur in either inert or non-inert gases (Table 1) [17]. In contrast, hydrothermal gasification is often conducted in the presence of supercritical water when temperature and pressure exceed its critical point (Table 1) [17]. On the other hand, non-thermal chemical methods operate at significantly lower temperatures compared to thermal chemical methods, typically below 150 °C. These methods involve direct reactions between plastic waste and chemical agents such as methanol, reducing overall energy consumption (Table 1). Pyrolysis or hydrolysis of plastic waste requires shorter reaction time and produces products with lower selectivity compared to hydrogenolysis and ammonolysis. Additionally, some biomass materials like *Enteromorpha clathrata*, cellulose, cooking oil, lignin, rice straw, sugarcane bagasse, and pine wood can undergo pyrolysis along with plastic waste and they can react together (Table 1). Photochemical methods offer another approach to plastic waste treatment. Unlike thermal methods, they do not require high temperatures, but rely instead on sunlight or ultraviolet (UV) radiation for the reaction process (Table 1). Harmless or useful small molecule products are produced after photocatalytic degradation of plastic waste. As shown in Table 1, a wide range of catalysts is employed to optimize plastic waste conversion. Pt/γ-Al_2_O_3_, MTO/Cl−Al_2_O_3_, electrocatalyst, choline chloride-2urea (ChCl-2Urea), stannous octoate, Pt@S-1, Pt/SrTiO_3_, Ir-^tBu^POCOP, [PdP(^t^Bu)_3_(m-Br)]_2_, commercial bentonite (CB), kaolin, silica gel, activated charcoal, composites with alumina-substituted Keggin tungstoborate (KAB) and kaolin, four Ni-Fe catalysts, MgO, Fe/Al_2_O_3_, HZSM-5 zeolite, Y-zeolite with transition metals, waste refinery catalyst, zeolite beta composite, CeO2-supported Ru, Ru-modified zeolite, ZSM-5, seawater, CaO/Fe_2_O_3_ oxygen carrier, Nb_2_O_5_, tetrabutylammonium decatungstate (TBADT), or Grubbs catalyst M_2_0_2_ are utilized as catalysts in the conversion process of plastic waste. The resulting products from these conversion processes vary and may include solids, liquids, or gases such as potassium diformate, terephthalic acid, H_2_, bisphenol A, PU, naphtha hydrocarbons, alkyl aromatics, C_2_–C_4_ olefins, 1,3-butadiene, C_4_–C_60_ *n*-paraffins, isoparaffins, mono-olefins, paraffins, naphthenes, aromatics, char, and carbon nanotubes (Table 1).

**Figure 1:**
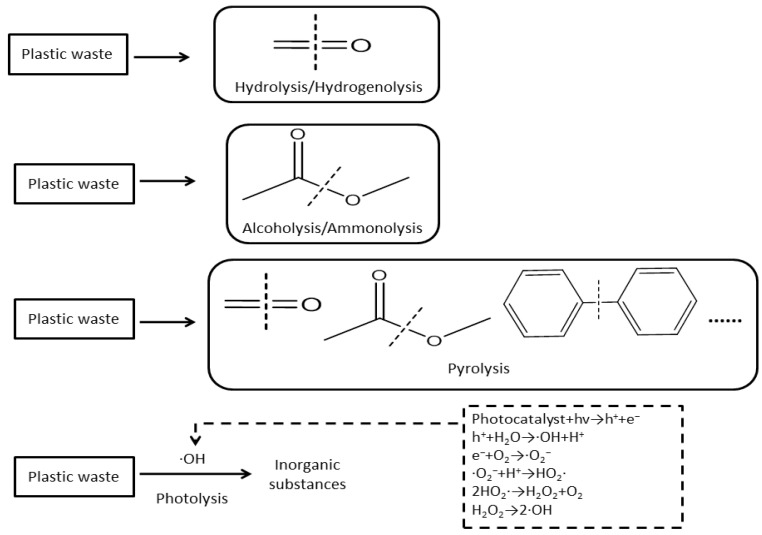
The mechanism of chemical conversion of plastic waste.

Hydrolysis, hydrogenolysis, alcoholysis, ammonolysis, pyrolysis, and photolysis are the fundamental reactions for the chemical conversion of plastic waste (Figure 1). These reactions break specific chemical bonds in polymers, such as carbon–carbon (C-C) or carbon–oxygen (C-O) bonds, to produce oligomers, monomers, or other small molecules (Figure 1). For example, hydrolysis and hydrogenolysis can target carbonyl groups in PC, while alcoholysis and ammonolysis primarily act on ester bonds (Figure 1) [21,55]. According to some researchers, C=C bonds were introduced from C_2_H_4_ to dehydrogenate PE [18,24]. For the pyrolysis process, the stability of molecular groups in plastic polymers varies across different temperature ranges, leading to the formation of diverse molecular groups and a wide variety of products. During the photolysis of plastic waste, photocatalysts absorb light energy and undergo electron transitions, forming electron–hole pairs. These pairs react with hydroxide ions to generate highly reactive hydroxyl radicals, which then oxidize the plastic waste into inorganic substances (Figure 1). However, the types of radicals formed may vary depending on the specific processes. The result obtained by Kong et al. [54] showed that electron–hole pairs could abstract a hydrogen atom from C-H bonds of PE to produce a long-chain alkyl radical, which formed an aminyl radical with the addition of DIAD. In a different approach, Zeng et al. [48] used UV light to promote the bromination of PE instead of its direct photolysis, presenting an alternative light-driven strategy. Carbonyl groups in polymers can induce Norris type I and type II reactions upon absorption of light [56]. Polymers containing carbonyl groups undergo Norris type I reactions and form acyl and alkyl radicals [56]. Carbonyl groups in polymers with gamma hydrogen undergo Norris type II reactions, where gamma hydrogen molecules transfer to oxygen to form 1,4-bis radicals [56].

**Figure 2:**
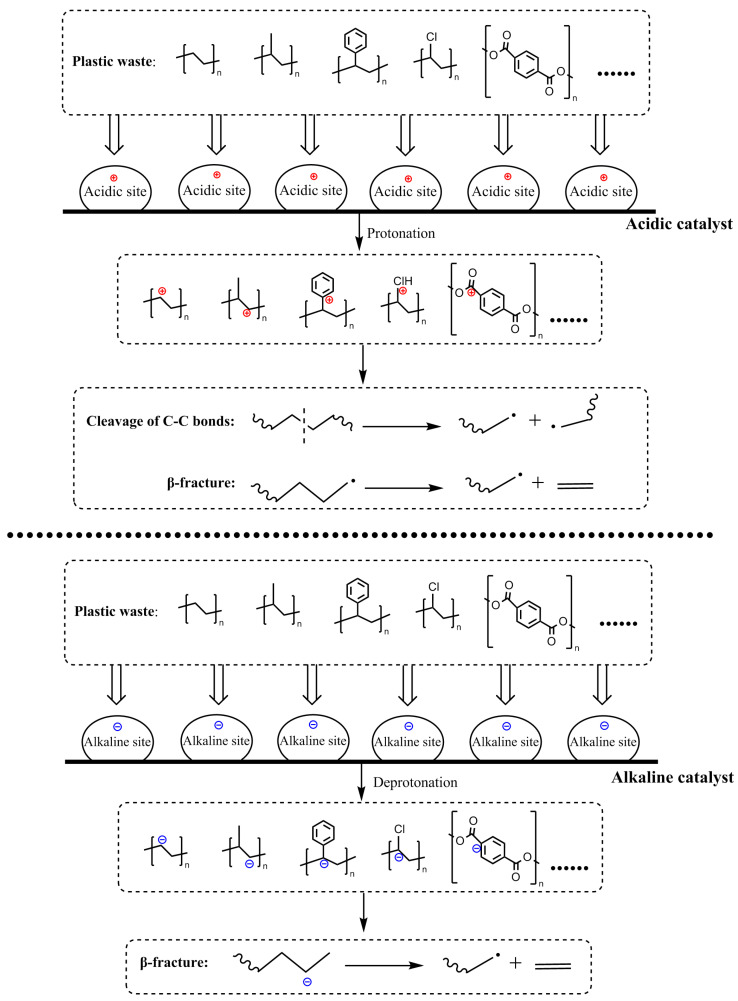
The catalytic mechanism of acidic and alkaline catalysts in the pyrolysis process.

Catalysts play a pivotal role in the chemical conversion of plastic waste, facilitating its efficient transformation into hydrocarbons with a narrow distribution by altering activation energy and regulating reaction kinetics. The pore structure and pH of the catalysts can significantly affect the catalytic performance. During pyrolysis, for instance, carbon-positive ions are generated through acid catalysis, thereby promoting the cleavage of C-C bonds in plastic polymers (Figure 2) [57]. Among various catalysts, zeolite molecular sieves, i.e., solid acid catalysts composed of Si/Al with well-ordered pores, exhibit unique catalytic activity towards C-C bond cleavage. During pyrolysis, long-chain hydrocarbons are initially produced, followed by β-fracture of the long polymer chains under the action of acidic sites in zeolite molecular sieves or other carbocations (Figure 2), ultimately yielding gas and liquid products with specific carbon distributions. Smaller zeolite molecular sieves can increase the heat transfer rate, reaction rate, and oil yield [58]. Furthermore, Xie et al. [59] reported that microporous structures favored small-molecule gas products such as ethylene and propylene, and mesoporous structures promoted macromolecular products such as aromatics. For the pH of zeolite molecular sieves, it was reported that the acidity of the catalyst was higher when the ratio of silicon and aluminum was lower, leading to higher yields of light gaseous hydrocarbons and liquid aromatic hydrocarbons [59]. Activated carbon, another effective catalyst, also relies on its acidity for catalytic activity. During its production, functional groups such as C=O and -OH are generated, forming Brønsted acid sites that facilitate the cleavage of C-C and C-H bonds, resulting in lighter hydrocarbons [57]. Simultaneously, dehydrogenation at Lewis sites promotes the aromatization of products [57]. Alkali metal oxides and transition metal oxides can also catalyze the pyrolysis process. They possess active alkaline sites that attack hydrogen atoms on polymer chains, forming carbon negative ions, which then undergo β-fracture to produce light hydrocarbons (Figure 2). Metal carbonates, which decompose into metal oxides with active alkaline sites upon heating, have been used to catalyze the pyrolysis of plastic polymers, enabling depolymerization through catalytic pyrolysis [60,61,62,63]. In addition to the carbocation mechanism, researchers have proposed that catalytic cracking of plastic waste followed the free radical mechanism [64]. Free radical initiation, chain reaction and termination reaction occurred in the pyrolysis process. Researchers have reported that an environment rich in H∙ was formed on the surface of the catalyst when hydrogen and Fe/AC were present in the system [64]. H∙ reacted with the free radicals produced by the cracking of polyethylene [64]. The pyrolysis of plastic waste often produces harmful aromatic compounds such as benzene, aniline, and their derivatives. To address this issue, researchers have used nickel catalysts to convert the hazardous chemicals produced from pyrolysis into value-added syngas [59].

**Figure 3:**
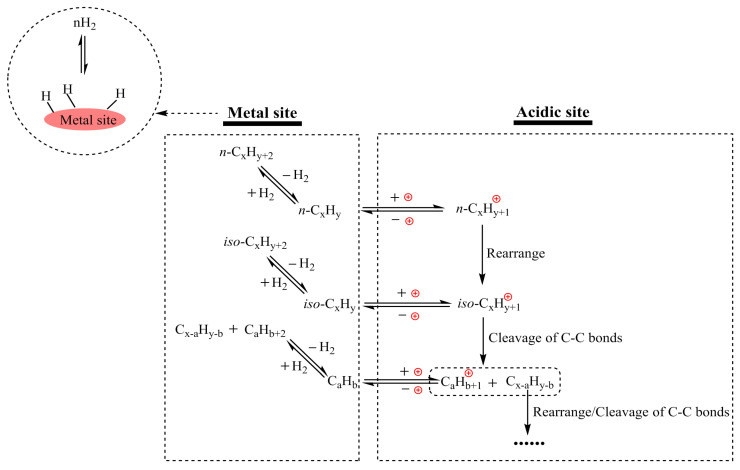
The catalytic mechanism of bifunctional catalysts in the pyrolysis process.

With continued progress in the field, recent research has shifted toward resolving issues such as carbonization and sintering, which pose significant threats to catalyst lifespan and performance. Introducing hydrogen into catalytic cracking has emerged as a promising strategy to minimize carbon deposition in catalysts while enhancing the yield and selectivity of gasoline and diesel fractions [57]. Hydrogenation pyrolysis usually employs bifunctional catalysts composed of metal and acidic sites (Figure 3) [59]. The metal active center promotes the dissociation of hydrogen molecules into active hydrogen atoms, while acidic sites facilitate the cleavage of C-C bonds (Figure 3). This combined effect enables the decomposition of plastic polymers into stable small-molecule hydrocarbons. Furthermore, the presence of hydrogen lowers the required pyrolysis temperature compared to non-hydrogenated systems, improving process efficiency (Table 1). Bifunctional catalysts are mainly divided into precious metal (Rh, Ru, Pt) and non-precious metal (Ni, Cu, Fe, Co, W) catalysts [59]. Researchers have reported that the cleavage of C-C bonds, the β-scission of alkylcarbenium ions, and skeletal rearrangements occurred with the assistance of strong Brønsted acidity of Rh/Nb_2_O_5_, promoting one-step solvent-free catalytic hydrogenolysis and isomerization of plastic polymers [65]. Additionally, reaction pressure plays a crucial role in the hydrogenation pyrolysis of plastic waste [59]. Increasing hydrogen pressure in the reaction system enhances the coverage of active hydrogen on catalyst surfaces, facilitating the hydrogenation saturation of intermediate products and their subsequent desorption from the catalyst surface. This process not only accelerates the reaction rate but also helps prevent excessive depolymerization and the overproduction of small-molecule gases while improving the release of products from the catalyst surface.

### 2.2. Biological Methods

Biological methods for treating plastic pollution include animal biodegradation and microbial biodegradation [66,67,68]. However, most research focuses on utilizing microorganisms to degrade plastic waste. These methods, commonly known as biodegradation, employ microorganisms or enzymes to convert high-molecular-weight macromolecules into oligomers or small molecules. Biodegradation typically involves either the assimilation of microorganisms or direct enzymatic degradation, which disrupts the structure of plastic polymers, reducing their molecular weight [69,70]. The efficiency of plastic biodegradation largely depends on its physical and chemical properties, with biodegradable and environmentally friendly plastic waste breaking down more readily [70,71,72,73,74]. Critical factors such as crystallinity, hydrophilicity, molecular weight, and toughness have a decisive impact on the biodegradability of plastic waste [16]. Plastic waste with higher crystallinity is more resistant to biodegradation than that with lower crystallinity [16]. The presence of functional groups in plastic polymers increases their hydrophilicity, thus enhancing their biodegradability [75]. Higher-molecular-weight plastics are less susceptible to degradation [16], and softer plastics degrade more quickly than harder ones [75]. Additionally, environmental factors such as moisture content, temperature, and pH influence microbial activity and enzyme efficiency, thus affecting biodegradation [76].

**Figure 4:**
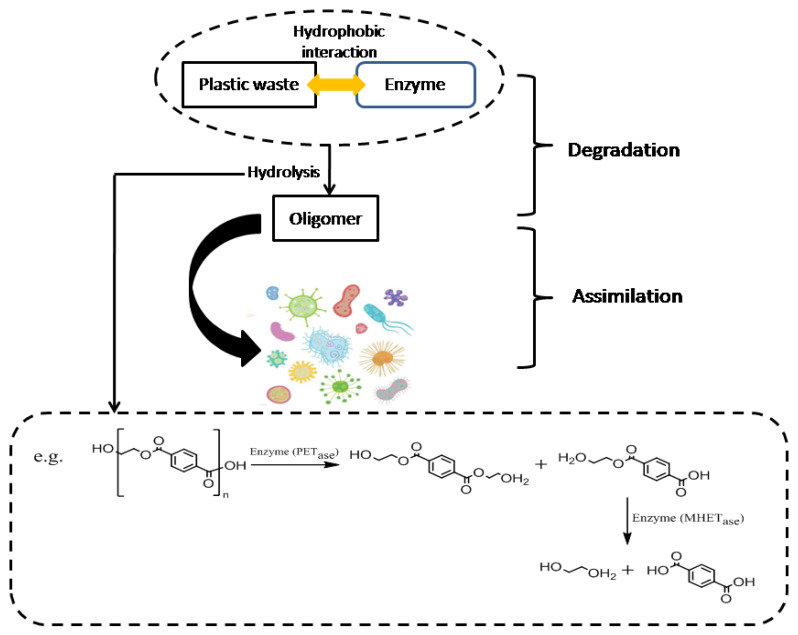
The mechanism of biodegradation of plastic waste.

The biodegradation of plastic waste involves two main mechanisms: degradation and assimilation (Figure 4). Microbial enzymes, either extracellular or intracellular, produced by microorganisms such as algae, bacteria, fungi, and actinomycetes are responsible for depolymerizing or decomposing plastic waste [77]. Among these enzymes, hydrolases play a crucial role by cleaving chemical bonds in the presence of water. When hydrolase acts on a product (A-B), the reaction typically follows Equation (1). Plastics found in the environment are generally hydrophobic [78]. The breakdown of plastic waste by hydrolases occurs in two steps. First, extracellular microbial enzymes adhere to the surface of plastic waste via hydrophobic interactions (Figure 4). The hydrophobic clefts in the active sites of these enzymes interact with hydrophobic groups on the plastic, improving the accessibility of the enzyme to the material. In the second step, the enzyme’s active sites hydrolyze specific chemical bonds within the plastic polymers, breaking them down into oligomers or small molecules that microorganisms can utilize as a carbon source (Figure 4).

(1) $$A-B+H_{2}O\to A-\mathrm{OH}+B-H$$

Recently, some research has focused on the discovery of novel plastic-degrading microorganisms. Numerous microbial species like *Thermobifida fusca*, *Serratia plymuthica* strain IV-11-34, *Pseudomonas aestusnigri*, *Pichia pastoris*, *Rhococcus* sp. *SSM1*, *Streptomyces scabies*, *Clostridium thermocellum*, *Pseudomonas citronellolis*, *Bacillus flexus*, *Aspergillus flavus*, *Cobetia* sp., *Halomonas* sp., *Exiguobacterium* sp., *Alcanivorax* sp., *Aspergillus flavus*, *Fusarium falciforme*, *Fusarium oxysporum*, *Purpureocillium lilacinum*, *Uronema africanum* Borge, *Stenotrophomonas* sp., *Achromobacter* sp., *Bacillus* spp., *Pseudomonas* spp., *Paenibacillus* sp., *Bacillus* sp., *Arthrobacter* sp., *Streptomyces* sp., *Sterigmatomyces halophilus*, *Meyerozyma guilliermondii*, *Meyerozyma caribbica*, *Enterobacter*, *Pseudomonas*, *Alcanivorax, Marinobacter*, *Arenibacter*, *Bacillus* spp., *Spirulina* sp., *Streptomyces* sp., *Phaeodactylum tricornutum*, *Chaetomium globosum*, and anaerobic marine consortia have been found to degrade plastic waste effectively (Table 2). Microorganisms are capable of degrading plastic produce hydrolases such as cutinase, lipase, esterase, and alkane monooxygenase, which facilitates the breakdown of plastic polymers into smaller molecules (Table 2). The biological conversion of plastic waste generally occurs at lower temperatures than chemical methods (Table 1 and Table 2) because high temperatures can deactivate the enzymes. In addition to the high temperature, light, humidity, and chemicals can also affect the growth and metabolism of microorganisms, impacting biomass and enzyme degradation activity negatively. However, the biological process often requires longer processing times (Table 2). Additionally, the hydrophobic nature of plastic polymers, attributed to their hydrocarbon chain structures, can hinder microbial activity during biodegradation. As a solution, thermal or UV pretreatment is frequently applied to polyolefin plastics, making them more amenable to biodegradation [78].

Bacteria are the main group of microorganisms used to degrade plastic waste. Plastic, as a type of carbon-based organic compound, can serve as a carbon source for the growth of bacteria. Under laboratory conditions, research on the degradation of plastic waste by bacteria mainly focuses on the use of pure bacterial cultures. These cultures are mostly isolated and enriched from soil, ocean, sludge, and even insect intestines [78]. Zhang et al. [109] isolated a bacterial strain (*Klebsiella* sp. EMBL-1) from the intestinal tract of larvae, and the bacterial strain could depolymerize and utilize PVC as the sole carbon source. The composite microbial communities may degrade plastic waste more effectively compared to pure bacterial strains because of collaborative symbiosis [110]. Skariyachan et al. [111] screened bacterial strains from waste treatment plants and combined them into a composite microbial community. They found that the new microbial community had higher potential for degrading plastic waste comparing to single strains [111]. The degradation efficiencies of the microbial community on low-density polyethylene films and particles were about 81% and 38%, respectively [111]. Although there is some research supporting that the degradation rate of plastic waste by complex bacterial communities is higher than single strains, the evidence is still very limited. Mixed bacteria require appropriate matching in order to have better degradation effects, while some competing bacteria may produce opposite effects.

Some research suggested that fungi might have a better degradation effect on plastic waste than bacteria, as fungal hyphae could adhere more firmly to the surface of plastic waste and might penetrate into the interior of particles compared to bacteria [112]. Furthermore, fungi could also promote the formation of chemical bonds such as carbonyl, carboxyl, and ester groups in plastics, thereby reducing their hydrophobicity [113]. The ability of fungi to degrade plastic waste was attributed to their enzyme system, which secreted lignin-modifying enzymes (LME) including manganese peroxidase (MnP), lignin peroxidase (LiP), multifunctional peroxidase, and laccase (Lac). These enzymes could break down and mineralize lignin ultimately. Polymers and lignin have similar chemical structures (i.e., ether bonds, aromatic rings, carbon skeleton, etc.), and thus LME can degrade plastic waste [114]. Researchers have found that lignin peroxidase secreted by *P. chrysosporium* can alter the structure of PVC [115].

**Figure 5:**
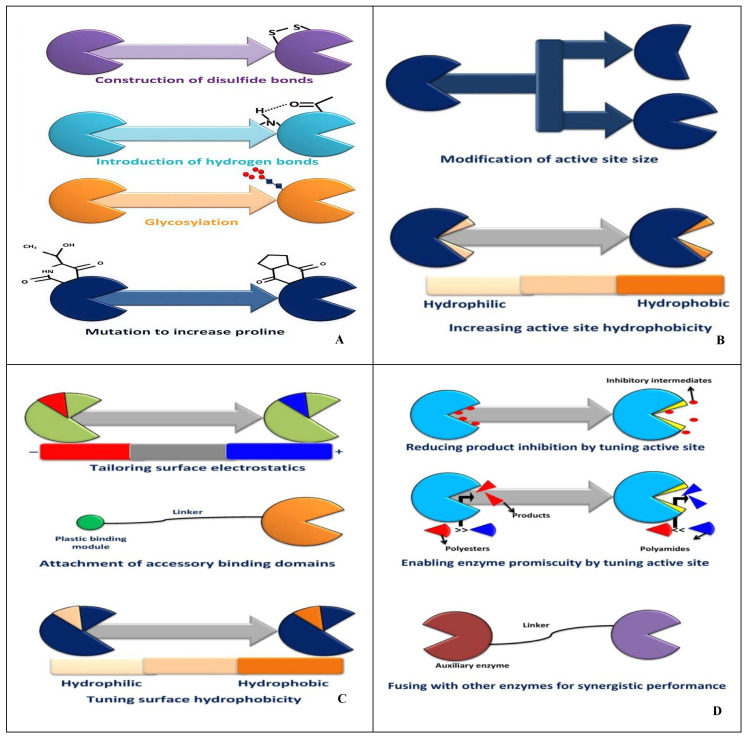
The mechanism of improving the performance of plastic-degrading enzymes. (**A**) Enhancing the thermal stability of enzymes; (**B**) enhancing the attachment of plastic waste to the active sites of enzymes; (**C**) enhancing the interaction between plastic waste and the surface of enzymes; (**D**) refining additional functions of enzymes (modified from [78]).

Plastic waste can be enzymatically degraded through cell-free or whole-cell biocatalysis [78]. This process begins with microbial fermentation under controlled conditions, including temperature, oxygen levels, pH, and nutrients [78]. In the cell-free approach, microbial cells are disrupted before enzyme extraction, while in the whole-cell process, plastic waste is incubated with high-density microbial cultures [78]. Enhancing the efficiency of plastic-degrading enzymes has been a key focus in protein engineering and synthetic biology. These improvements primarily target four main areas: (1) enhancing the thermal stability of enzymes; (2) improving the attachment of plastic waste to the active sites of enzymes; (3) strengthening the interactions between plastic waste and the surface of enzymes; and (4) refining additional functions of enzymes (Figure 5). The thermal stability of plastic-degrading enzymes can be promoted via adding disulfide bonds or salt bridges (Figure 5A) [116]. Enzymes depend on disulfide bonds or salt bridges to fold into a local or global shape, which is beneficial for improving heat resistance. For example, a disulfide bond created by the TfCut2 esterase from *Thermobifidafusca* and a salt bridge connecting Arg280 and N246D residues could increase the temperature at which the proteins break down [78]. Another strategy to enhance the thermal stability of enzymes is to engineer the creation of hydrogen bonds in the region (Figure 5A) [117], which can preserve higher-order protein structures of enzymes and make the structure of enzymes more stable. For example, the thermal stability of PETase can be greatly promoted by a hydrogen bond created through water between S121E and N172 residues [78]. Additionally, glycosylation enhances the thermal stability of enzymes by strengthening the thermodynamic stabilization of enzymes and preventing the thermal aggregations of enzymes (Figure 5A) [117]. For example, glycosylated LCC showed higher thermal stability and higher PET hydrolysis activity at elevated temperatures [117]. Furthermore, the cyclic structure of the proline side chain can reduce the conformational entropy opposing protein folding, which can make the structural rigidity higher. Therefore, introducing more proline residues is beneficial to enhance the thermal stability of enzymes (Figure 5A). For example, threonine was mutated into proline at the 235 position of a *Thermobifida alba* cutinase, and PETase had increased hydrolytic activity [117]. A common strategy to improve the attachment of plastic waste to the active sites of enzymes is creating a wider opening of the active sites to increase the accessibility of plastic substrate (Figure 5B). However, a wider opening of the active sites does not always show improved catalytic performance, as an overly enlarged active site may lead to weaker substrate affinity because of reduced binding ability [117]. In certain situations, modifying the active site with a narrower space is favorable (Figure 5B). Also, the hydrophobicity of the enzyme binding groove of active sites is a potential engineering target (Figure 5B). Increasing the hydrophobicity can be conducive to plastic binding resulting from higher affinity. For instance, PETase can bind to plastic substrates and degrade PET more easily after increasing the hydrophobicity [78]. The substrate-binding process is affected by electrostatic and hydrophobic interactions between plastic polymers and amino acid residues on the surface of enzymes. Hence, tailoring surface electrostatics and tuning surface hydrophobicity are common strategies (Figure 5C). Specifically, making the surface of enzymes electrically neutral can reduce electrostatic repulsion between plastic waste and enzymes, thus promoting the degradation efficiency of plastic waste. Another method to enhance the interaction between plastic waste and the surface of enzymes is the attachment of accessory binding domains to the surface of enzymes (Figure 5C). This approach is inspired by the fact that some enzymes show an auxiliary binding domain specialized in substrate adhesion. Therefore, plastic-degrading enzymes’ absence of such function can be fused with heterologous binding modules to promote interaction with plastic waste. For instance, enzymes catalyzing the breakdown of carbohydrates can produce carbohydrate-binding modules (CBMs) that can bind to many plastic polymers [78]. Efforts in optimizing the performance of enzymes have also been made in other aspects such as reducing product inhibition by tuning active sites, enabling enzyme promiscuity, and creating multifunctional biocatalysts (Figure 5D). First, intermediates or products in the degradation of plastic waste can inhibit the activity of enzymes, and such inhibition can be mitigated by tuning the active-site architecture [118]. For example, the G62A mutation in the substrate-binding groove of TfCut2 resulted in a 5.5-fold decrease, improving the degradation of PET [78]. Enabling enzyme promiscuity by tuning active sites is a meaningful approach to expand the degradation capacity of enzymes, especially for plastic waste that few known enzymes can efficiently degrade [118]. For example, synthesized PAs could be depolymerized by a cutinase called Thc_Cut1, increasing the activity of promiscuous amidase [78]. In addition, fusing with other enzymes for synergistic performance has been exploited to create bifunctional biocatalysts that can degrade plastic waste more efficiently [118]. For example, a fusion protein composed of lipase and cutinase (Lip-Cut) was developed, which could hydrolyze PCL more easily [78]. Research on protein engineering has mainly focused on PET-degrading enzymes, and the enzymes that can degrade other plastic waste efficiently are yet to be identified [119]. A logical chain of PETase activity towards PET is as follows: (1) PETase acts on ester bonds in PET and makes them break (hydrolysis); (2) mono(2-hydroxyethyl)terephthalate acid (MHET) is produced; (3) MHETase continues to act on ester bonds in MHET and makes them break (hydrolysis); (4) glycol and terephthalic acid are produced.

## 3. Economic Analysis and Life Cycle Assessment of Emerging Technologies

Economic analysis (EA) involves evaluating, comparing, and demonstrating the financial benefits of different technologies [120]. The costs associated with emerging technologies for treating plastic waste include raw materials, labor, equipment, and operations. For instance, Chhabra et al. [121] reported that the total cost of the pyrolysis equipment was USD 6.62 million, with reactors accounting for 48% of the total cost. According to a report by Hu et al. [14], the cost of thermal chemical methods is higher than other emerging methods. Promoting the valorization of organic waste, including plastic waste, is essential for fostering a circular economy [122,123,124,125]. Products derived from plastic waste treatment, such as bio-oil, biochar, and syngas, can be reused as energy sources [126,127,128]. For example, in a facility processing 200 tons of municipal solid waste daily, including PET, PE, and PP, bio-oil accounted for 86.8% of the total annual revenues of USD 11.53 million [121]. Beyond energy applications, other byproducts have significant industrial value. Carbon nanotubes, for example, have been used to manufacture produce transparent, conductive thin films. Similarly, terephthalic acid produced from the biological conversion of PET has been successfully utilized to synthesize PET bottles with mechanical properties comparable to those of petrochemically derived versions [88]. Such reuse of products not only offsets costs but also generates economic profits. To some extent, pyrolyzing waste plastics into fuels (such as gasoline and gaseous products for energy generation through combustion) can contribute to a circular economy. However, pyrolysis aimed at depolymerizing waste plastics into their original monomers offers a more promising route to achieving circularity.

Life cycle assessment (LCA) is a method of summarizing and evaluating all inputs and outputs of a system throughout its life cycle, as well as their potential impact on the environment [129]. Chhabra et al. [121] reported that the impact categories of oil production from plastic waste include acidification, climate change, freshwater ecotoxicity, freshwater eutrophication, human toxicity, land use, marine eutrophication, ozone depletion, and resource depletion. The potential impact on the environment (E_pot_) is the difference between the net environmental impacts of the benchmark waste treatment (EI_WT,_ n_et_) and ideal waste recycling (EI_WR,_ i_deal,_ n_et_) [129]. The net environmental impacts of the benchmark waste treatment (EI_WT,_ n_et_) are defined as direct environmental impacts of the benchmark waste treatment (EI_WT,_ d_irect_) minus the credit for avoided products (EI_avP_). The net environmental impacts of ideal waste recycling (EI_WR,_ i_deal,_ n_et_) are defined as direct environmental impacts of ideal waste recycling (EI_WR,_ i_deal,_ d_irect_) minus the credit for avoided chemicals (EI_avC_) [129]. The direct environmental impact of the benchmark waste treatment (EI_WT,_ d_irect_) consists of all environmental impacts required to treat 1 kg of plastic waste. Avoided environmental impacts (EI_avP_) include the credit for avoided products. Waste recycling converts 1 kg of treated plastic waste into m_j_ kg of chemicals. These chemicals substitute their conventional production and corresponding environmental impacts (EI_j_), leading to the credit for avoided chemicals (EI_avC_) [129]. Environmental impacts (EI_j_) are based on data from the LCA database. A method based on stoichiometry and thermodynamic data can be used to calculate EI_WR,_ i_deal,_ d_irect_ [129]. The method contains reactants ($\sum_{1}^{i}{\mathrm{EI}}_{i}m_{i}$), residual waste ($\sum_{1}^{k}{\mathrm{EI}}_{k}m_{k}$), and thermal energy (EI_H_Q_H_). m_i_ and m_k_ represent the mass of reactants and residual waste. EI_i_ and EI_k_ quantify the environmental impacts per kilogram, with EI_i_ representing the impact of reactant production and EI_k_ corresponding to the impact of residual waste treatment. EI_H_ represents the environmental impact of providing 1 MJ of energy using natural gas as fuel. Q_H_ represents the minimal energy demand for complete recycling per 1 kg of plastic waste. The relevant calculation formulas are as follows:

(2) $$E_{\mathrm{pot}}={\mathrm{EI}}_{\mathrm{WT},\;\mathrm{net}}-{\mathrm{EI}}_{\mathrm{WR},\;\mathrm{ideal},\;\mathrm{net}}$$

(3) $${\mathrm{EI}}_{\mathrm{WT},\;\mathrm{net}}={\mathrm{EI}}_{\mathrm{WT},\;\mathrm{direct}}-{\mathrm{EI}}_{\mathrm{avP}}$$

(4) $${\mathrm{EI}}_{\mathrm{WR},\;\mathrm{ideal},\;\mathrm{net}}={\mathrm{EI}}_{\mathrm{WR},\;\mathrm{ideal},\;\mathrm{direct}}-{\mathrm{EI}}_{\mathrm{avC}}$$

(5) $${\mathrm{EI}}_{\mathrm{avC}}=\sum_{1}^{j}{\mathrm{EI}}_{j}m_{j}$$

(6) $${\mathrm{EI}}_{\mathrm{WR},\;\mathrm{ideal},\;\mathrm{direct}}=\sum_{1}^{i}{\mathrm{EI}}_{i}m_{i}+\sum_{1}^{k}EI_{k}m_{k}+EI_{H}Q_{H}$$

**Figure 6:**
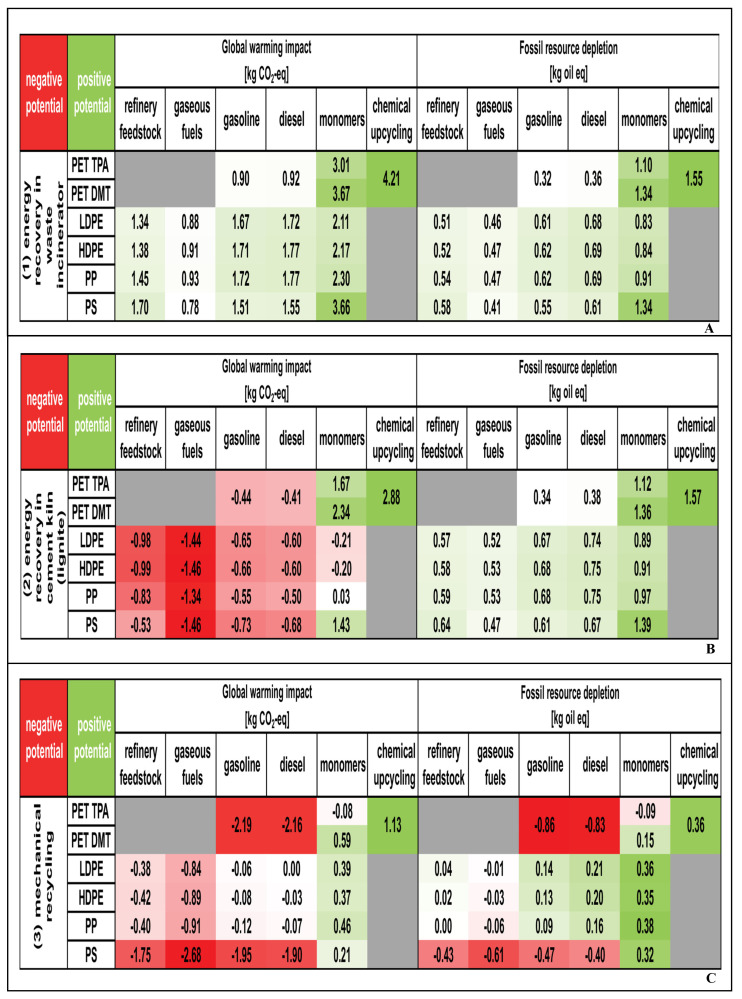
Comparison of the environmental potential of chemical recycling and other recycling methods. (**A**) Energy recovery in municipal solid waste incinerators; (**B**) energy recovery in cement kilns; (**C**) mechanical recycling (Modified from [129]). Red represents negative environmental potential. Green represents positive environmental potential. White represents values equal to zero. Gray represents that chemical recycling has been omitted.

According to Meys et al. [129], PET, PE, PP, and PS should not be chemically recycled into refinery feedstock or fuel products. Instead, mechanical recycling or utilization in cement kilns is recommended to reduce global warming impacts. Conversely, chemical recycling into monomers or value-added products could potentially reduce global warming impacts compared to energy recovery in municipal solid waste incinerators, energy recovery in cement kilns, and mechanical recycling (Figure 6). The environmental potential for global warming varies, ranging from 0.78 kg CO_2_-eq for producing gaseous fuels from PS to 4.21 kg CO_2_-eq for the chemical upcycling of PET (Figure 6A). Recycling plastic waste to refinery feedstock and fuels shows a negative environmental potential, from −1.46 kg CO_2_-eq for producing gaseous fuels from HDPE or PS to −0.44 kg CO_2_-eq for producing gasoline from PET (Figure 6B). Additionally, the environmental potential for PET and PS is negative in Figure 6C.

## 4. Future Perspectives

The use and implementation of emerging technologies for treating plastic waste face several significant challenges, including technological limitations, economic feasibility, scalability, environmental impacts, and regulatory constraints, which significantly impede their practical implementation and widespread adoption. Plastic waste feedstock is often complex, which makes the theoretical technical parameters of various emerging technologies not applicable. More in-depth research is needed on the impact of various factors on chemical or biological conversion of plastic waste. The impact and mechanism of each factor on each step of the conversion reaction need to be clarified. There are more factors in engineering applications than in laboratory research. How to identify the influencing factors in engineering applications is a future research direction. Fast catalyst deactivation caused by undesirable reactions is not conducive to engineering applications. The lack of sufficient research on the economic analysis and life cycle assessment of actual engineering is a problem. Additionally, contaminants (e.g., benzene, aniline, and their derivatives, chlorine, bromine, sulfur, and nitrogen in plastic oil and its quality standardization) are often produced in the thermal chemical conversion of plastic waste. In addition, financial barriers, uncompetitive marketing strategies, limited availability of quality plastic waste, gaps in plastic waste supply and demand, inefficient and costly plastic waste segregation technologies, and lack of local expertise in plastic waste recycling and ambiguous legislations also impede the large-scale commercial implementation of treating plastic waste by emerging technologies.

Currently, most research mainly uses a single method (chemical or biological) to treat plastic waste. However, both approaches have their distinct advantages and disadvantages. Integrating emerging chemical and biological methods to treat plastic waste may achieve complementary effects, making it a promising area for future research. While laboratory studies predominantly focus on single-type plastic waste, real-world scenarios often involve mixed plastic waste alongside other household waste. Therefore, future research should prioritize the treatment of mixed waste streams using emerging technologies, with particular attention on how non-plastic waste impacts the treatment process. Additionally, the technical parameters of various emerging technologies ought to be thoroughly validated for practical engineering applications to ensure their effectiveness and scalability. A core aspect of the technical parameters is the catalyst, and identifying high-performance catalysts is essential and urgent. Although pyrolysis, gasification, and hydrothermal gasification have been extensively studied for plastic waste treatment, research on hydrothermal carbonization and hydrothermal liquefaction remains limited. These methods present a promising direction, as they can operate under relatively mild temperature conditions compared to traditional thermal processes. Similarly, the photolysis of plastic waste remains underexplored, warranting further investigation to advance its application in plastic waste degradation. The mechanisms underlying the hydrothermal modification of plastic waste also require deeper study to enhance process efficiency. In biodegradation studies, microorganisms are predominantly used to treat plastic waste. However, plants and small animals also play a role in pollutant absorption. Future research should explore whether these biological systems can be leveraged for plastic waste degradation while assessing the potential adverse effects of plastic pollution on these organisms. Additionally, current protein engineering research has mainly focused on PET-degrading enzymes, leaving a significant gap in identifying enzymes capable of efficiently degrading other types of plastic waste. Furthermore, studies on the EA and LCA of emerging plastic waste treatment technologies remain insufficient. More case studies are essential to comprehensively assess the environmental and economic feasibility of these methods, along with further research to refine and validate the applicability of LCA calculation formulas in real-world engineering applications. While many byproducts from emerging plastic waste treatment technologies show potential for reuse, additional research is necessary to validate their practical applications. Advancing these research efforts will be essential for addressing challenges in plastic waste management and maximizing the potential of emerging technologies.

## 5. Conclusions

Emerging technologies for converting and utilizing plastic waste mainly include chemical and biological technologies. Chemical methods focus on breaking down high-molecular-weight macromolecules into oligomers or small molecules by cracking or depolymerizing chemical bonds in plastic polymers. Key reactions for chemical conversion include hydrolysis, hydrogenolysis, alcoholysis, ammonolysis, pyrolysis, and photolysis, which cleave specific bonds in plastic polymers to produce oligomeric products. Catalysts are crucial in these processes, as they lower activation energy, regulate reaction kinetics, and facilitate the conversion of plastic waste into hydrocarbons with narrow distributions. Factors such as pore structure and pH of catalysts can significantly affect their performance. However, issues like carbonization and sintering can reduce catalyst efficiency and lifespan. Introducing hydrogen into catalytic cracking can not only effectively solve the problem of carbon deposition in catalysts but also improve the yield and selectivity of gasoline and diesel fractions. Biological methods generally involve biodegradation, where microorganisms or enzymes break down macromolecules into oligomers or small molecules. The mechanism of biodegradation of plastic waste is degradation and assimilation. Chemical conversion of plastic waste requires less time and is more thorough compared to biological technologies. Chemical technologies are suitable for converting and utilizing most types of plastic waste; however, biological technologies are more suitable for converting and utilizing plastic waste with lower crystallinity. From the economic perspective, the cost of chemical technologies is generally higher than biological technologies. The economic analysis of emerging plastic waste treatment technologies involves calculating, comparing, and evaluating different methods, serving as a key tool for selecting the most cost-effective solutions. Life cycle assessment evaluates the inputs, outputs, and potential environmental impacts of emerging plastic waste treatment technologies across the entire life cycle of a system, guiding sustainable decision-making and process optimization. In summary, significant research is necessary to address the challenges and optimize the conversion and utilization of plastic waste through emerging technologies, ensuring their scalability, efficiency, and sustainability.

## Figures and Tables

**Table 1:** Technical parameters of chemical methods for converting or recycling plastic waste.

Plastic Waste	Device	Reactant	Catalyst	Temperature	Reaction Medium	Illumination	Product	Reference
PE PET PC PC PE PE PE PE PE Plastic mixture Plastic mixture Low-density PE PP PE PS PVC High-density PE PP PET Medical masks Plastic mixture Low-density PE Plastic mixture Plastic mixture Low-density PE Plastic mixture PE, PP PE, PC, PP, ABS PS PP PE Plastic mixture PE PE Low-density PE PE PP, PS PE PE	Tank reactor Electrolyzer Autoclave Reaction vessel Autoclave Autoclave Reaction vessel Autoclave Furnace Fixed bed reactor Tube reactor Fixed bed reactor Fixed bed reactor Fixed bed reactor Fixed bed reactor Fixed bed reactor Fixed bed reactor Autoclave Horizontal furnace Tube furnace Autoclave Autoclave Autoclave Microwave oven Microwave oven Microwave oven Microwave oven Autoclave Tube reactor Tube reactor Fixed bed reactor Reaction vessel Autoclave Fluidized bed reactor Autoclave Pyrolyzer Microwave oven Fluidized bed gasifier, tar-cracking reactor Reaction vessel	C_2_H_4_ H_2_O Methanol C_6_HN, C_8_HN_2_O_2_ / / C_2_H_4_ / / / / / / / / EC / / / / / / / Cooking oil Lignin / / Soda lignin / / Pine wood / Br_2_, ethylene / / Cellulose Rice straw, sugarcane bagasse / DIAD	Pt/γ-Al_2_O_3_, MTO/Cl−Al_2_O_3_ Electrocatalyst ChCl-2Urea Stannous octoate Pt@S-1 Pt/SrTiO_3_ Ir-^tBu^POCOP, [PdP(^t^Bu)_3_(m-Br)]_2_ Pt/γ-Al_2_O_3_ KAB/kaolin composites Four Ni-Fe catalysts Activated carbon Activated carbon, MgO Fe/Al_2_O_3_ Fe/Al_2_O_3_ Fe/Al_2_O_3_ / Y-zeolite with transition metals Waste refinery catalyst / / Zeolite beta composite CeO_2_-supported Ru Ru-modified zeolite / / / ZSM-5 / / Seawater CaO/Fe_2_O_3_ oxygen carrier Nb_2_O_5_ Grubbs catalyst M202 / CB, kaolin, silica gel, activated charcoal HZSM-5 zeolite HZSM-5 Active carbon TBADT	100 °C 60 °C 130 °C 70–75 °C 250 °C 300 °C 130–350 °C 280 °C 295 °C 500 °C 430–571 °C 450–600 °C 500 °C 500 °C 500 °C 550 °C 600 °C 100–450 °C 600–1000 °C 900 °C 360–400 °C 200 °C 300 °C 400–550 °C 550 °C 450–500 °C 500–740 °C 500–750 °C 500–800 °C 500–800 °C 750–850 °C RT 30–105 °C 500–600 °C 550–650 °C 650 °C 500 °C 790–840 °C 110 °C	Atmospheric C_2_H_4_ KOH aqueous solution Autogenous pressure Anisole 3 MPa of H_2_ 170 Pa of H_2_ / / N_2_ N_2_ N_2_ N_2_ N_2_ N_2_ N_2_ N_2_ N_2_ / N_2_ Ar 20 bar of H_2_ 2 MPa of of H_2_ 50 bar of H_2_ Negative pressure N_2_ / / Supercritical water Supercritical water Supercritical water N_2_ / 2.7 bar of ethylene N_2_ / N_2_ N_2_ Air or oxygen /	/ / / / / / / / / / / / / / / / / / / / / / / / / / / / / / / Sunlight 400−410 nm UV / / / / / Sunlight	Propylene Potassium diformate, terephthalic acid, H_2_ Bisphenol A PU Naphtha hydrocarbons Fuel oil Propylene Alkylaromatics, alkylnaphthenes Fuel oil, syngas Carbon nanotubes Jet fuel, H_2_-enriched gases Jet fuel, H_2_-enriched gases H_2_, liquid fuels, carbon nanotubes H_2_, liquid fuels, carbon nanotubes H_2_, liquid fuels, carbon nanotubes Bio-oil, bio-char, non-condensable gas Aromatic fuel oils, H_2_ Methylbenzenes, alkanes Porous carbon Porous carbon materials Gasoline Liquid fuels, waxes CH_4_ Liquid fuel Hydrocarbon rich bio-oil Fuel oil Fuel oil Syngas H_2_, CH_4_, CO_2_ H_2_, CH_4_, CO_2_ Syngas with high H_2_/CO ratio C_2_ fuels *α*,*ω*-divinyl-functionalized oligomer H_2_, C_1_–C_4_ paraffins, C_2_–C_4_ olefins, 1,3-butadiene, C_4_–C_60_ *n*-paraffins, isoparaffins, mono-olefins, cycloalkanes/alkadienes, aromatics Paraffins, isoparaffins, olefins, naphthenes, aromatics, char, syngas Oxygenated chemicals, olefins, alkanes, aromatics Bio-oil, biochar, gas Syngas, tar Low molecular weight PE with tunable polarity	[18] [19] [20] [21] [22] [23] [24] [25] [26] [27] [28] [29] [30] [30] [30] [31] [32] [33] [34] [35] [36] [37] [38] [39] [40] [41] [42] [43] [44] [45] [46] [47] [48] [49] [50] [51] [52] [53] [54]

Abbreviations: ChCl-2Urea: choline chloride-2urea; KAB: alumina-substituted Keggin tungstoborate; EC: *Enteromorpha clathrata*; RT: room temperature; UV: ultraviolet; CB: commercial bentonite; DIAD: diisopropyl azodicarboxylate; TBADT: tetrabutylammonium decatungstate.

**Table 2:** Technical parameters of biological methods for converting or recycling plastic waste.

Plastic Waste	Microorganism/Enzyme	Reaction Condition	Product	Reference
PET PET PET PET PET PET PET PET PET PET PET PVC PVC PVC PP PE PE PE PE PE PE PE PE PE PE PE PE, PP PE, PET PE, PVC PP, PET	*Thermobifida fusca*/cutinase (TfCut2) *Serratia plymuthica* strain IV-11-34/synthase *Pseudomonas aestusnigri*/carboxylic ester hydrolase *Pichia pastoris*/PETase *Rhococcus* sp. *SSM1*/PETase *Streptomyces scabies*/protein sub1 *Clostridium thermocellum*/thermophilic cutinase *Streptomyces* sp. *Phaeodactylum tricornutum*/PETase LCC–ICCG variant/Depolymerase *Bacillus subtilis* HR29/BhrPETase *Pseudomonas citronellolis*, *Bacillus flexus* *Chaetomium globosum* Anaerobic marine consortia *Aspergillus* sp., *Penicillium* sp. *Aspergillus flavus*/AFLA_006190, AFLA_053930 *Cobetia* sp., *Halomonas* sp., *Exiguobacterium* sp., *Alcanivorax* sp. *Aspergillus flavus*, *Fusarium falciforme*, *Fusarium oxysporum*, *Purpureocillium lilacinum* *Uronema africanum* Borge *Stenotrophomonas* sp., *Achromobacter* sp./cutinase, lipase, esterase, alkane monooxygenase *Bacillus* spp., *Pseudomonas* spp. *Paenibacillus* sp., *Bacillus* sp. *Arthrobacter* sp., *Streptomyces* sp. *Sterigmatomyces halophilus*, *Meyerozyma guilliermondii*, *Meyerozyma caribbica*/MnP, Lac, LiP *Enterobacter cloacae* AKS7 PE-degrading bacteria, PHA-synthesizing bacteria *Enterobacter*, *Pseudomonas* *Alcanivorax*, *Marinobacter*, *Arenibacter* *Bacillus* spp. *Spirulina* sp.	1000 r/min, 70 °C, 96 h 26 °C, 30 d 30 °C, 48 h 30 °C, 18 h 34 °C, pH 8.5 37 °C, 20 d Anaerobically, 60 °C, 14 d 120 rpm, 28 °C, 18 d 21–30 °C, 180 d 65 °C, 14 h, pH 8 37 °C, pH 7 Aerobically, 30 d 28 °C, 28 d Anaerobically, 20 °C, 2 a 29 °C, 30 d 28 d 30–90 d 30 d 30 d Aerobically, 150 rpm, 30 °C, 45 d 30 °C, 30 d 30 °C, 60 d 120 r/min, 25 °C, 90 d 30 °C, 45 d 30 °C, 45 d 30 °C, 21 d 37 °C, 160 d 30 °C, 80 d 180 rpm, 30 °C, 90 d 112 d	Ethylene glycol, terephthalic acid Small molecules Bis(2-hydroxyethyl) terephthalate, mono(2-hydroxyethyl) terephthalate Small molecules Small molecules Terephthalic acid Small molecules Small molecules Terephthalic acid, mono(2-hydroxyethyl) terephthalic acid Small molecules Small molecules Small molecules Small molecules Small molecules Small molecules Small molecules Small molecules Small molecules Small molecules Small molecules Small molecules Small molecules Small molecules Small molecules Small molecules Small molecules Small molecules Small molecules Small molecules Small molecules	[79] [80] [81] [82] [83] [84] [85] [86] [87] [88] [89] [90] [91] [92] [93] [94] [95] [96] [97] [98] [99] [100] [101] [102] [103] [104] [105] [106] [107] [108]

## Data Availability

No new data were created or analyzed in this study. Data sharing is not applicable to this article.
